# Automatic ICD-10 multi-class classification of cause of death from plaintext autopsy reports through expert-driven feature selection

**DOI:** 10.1371/journal.pone.0170242

**Published:** 2017-02-06

**Authors:** Ghulam Mujtaba, Liyana Shuib, Ram Gopal Raj, Retnagowri Rajandram, Khairunisa Shaikh, Mohammed Ali Al-Garadi

**Affiliations:** 1 Department of Information Systems, Faculty of Computer Science and Information Technology, University of Malaya, Kuala Lumpur, Malaysia; 2 Department of Computer Science, Sukkur Institute of Business Administration, Sukkur, Pakistan; 3 Department of Artificial Intelligence, Faculty of Computer Science and Information Technology, University of Malaya, Kuala Lumpur, Malaysia; 4 Department of Surgery, Faculty of Medicine, University of Malaya, Kuala Lumpur, Malaysia; 5 Department of Community Medicine, Shaheed Mohtarma Benazir Bhutto Medical University, Larkana, Pakistan; Nanjing Normal University, CHINA

## Abstract

**Objectives:**

Widespread implementation of electronic databases has improved the accessibility of plaintext clinical information for supplementary use. Numerous machine learning techniques, such as supervised machine learning approaches or ontology-based approaches, have been employed to obtain useful information from plaintext clinical data. This study proposes an automatic multi-class classification system to predict accident-related causes of death from plaintext autopsy reports through expert-driven feature selection with supervised automatic text classification decision models.

**Methods:**

Accident-related autopsy reports were obtained from one of the largest hospital in Kuala Lumpur. These reports belong to nine different accident-related causes of death. Master feature vector was prepared by extracting features from the collected autopsy reports by using unigram with lexical categorization. This master feature vector was used to detect cause of death [according to internal classification of disease version 10 (ICD-10) classification system] through five automated feature selection schemes, proposed expert-driven approach, five subset sizes of features, and five machine learning classifiers. Model performance was evaluated using precision_M_, recall_M_, F-measure_M_, accuracy, and area under ROC curve. Four baselines were used to compare the results with the proposed system.

**Results:**

Random forest and J48 decision models parameterized using expert-driven feature selection yielded the highest evaluation measure approaching (85% to 90%) for most metrics by using a feature subset size of 30. The proposed system also showed approximately 14% to 16% improvement in the overall accuracy compared with the existing techniques and four baselines.

**Conclusion:**

The proposed system is feasible and practical to use for automatic classification of ICD-10-related cause of death from autopsy reports. The proposed system assists pathologists to accurately and rapidly determine underlying cause of death based on autopsy findings. Furthermore, the proposed expert-driven feature selection approach and the findings are generally applicable to other kinds of plaintext clinical reports.

## 1.0 Introduction

Autopsy or postmortem examination provides useful contribution to health-related education and improves the quality of the healthcare industry [1,2]. In autopsy examination, medical experts called pathologists to examine the dead body externally and internally and collect information related to external and internal body organs. Pathologists also collect information regarding personal details, injury, histopathology reports, and previous medical history of the deceased person [3]. The collected autopsy findings are correlated with medical history, premortem and postmortem laboratory studies, microscopic findings of tissues, toxicology, and other related medical procedures and documents to determine and assign the cause of death according to the World Health Organization internal classification of disease version 10 (ICD-10) coding standard [4]. Thus, performing autopsy is both laborious and time consuming.

Autopsy examination culminates in the generation of autopsy reports. The two main types of autopsy reports include clinical/pathological and forensic (medico-legal) [5]. In developing countries, a third type of autopsy report, namely, verbal autopsy, is available [6]. Clinical autopsy is performed to discover the medical cause of death. Clinical autopsy is usually conducted in situations of uncertain deaths. Thus, preventive actions should be carried out to avoid such incidents in future. Forensic autopsy is performed to discover the cause of death in criminal matter [5]. In verbal autopsy, an interview is conducted from the relatives or witnesses of the deceased person to discover the cause of death. This method is common in low economical countries, where health facilities are insufficient [6].

Autopsy examination improves the quality of healthcare. Many hospitals used electronic database systems, in which autopsy findings and reports are stored in a free text format. In several situations, experts use and correlate these stored autopsy reports to solve future cases. However, the greatest challenges in performing autopsy are lack of human resources and insufficient time during the investigation to determine the cause of death [7]. Therefore, these reports must be converted into actionable information that can be used by pathologists to accurately and rapidly determine the cause of death. Various intelligent systems have been developed to identify and contextualize concepts of interests, also called named entities, from plaintext clinical reports by using automatic text classification techniques (ATC). ATC is an automated process of assigning set of predefined categories to plaintext documents [8,9]. In recent years, machine learning and ATC techniques have been applied in various application areas such as banking and finance [10,11], e-commerce systems [12] and bioinformatics [13,14]. ATC is commonly performed using either ontology-based ATC technique or supervised ATC technique [9,15].

In ontology-based ATC technique, domain-related medical ontologies are developed and used to identify named entities. To classify pathology reports as cancer positive or negative by using ontology-based ATC technique, we first created an ontology related to cancer identification. This cancer-related ontology includes a set of concepts namely, things, events, or things, which are specified to develop an agreed-upon vocabulary for information exchange [8,9,15]. Ontology-based ATC techniques can utilize suitable entities that imitate the concept of interest. However, medical ontologies are lacking to imitate entities for certain condition or disease. Moreover, extensive domain knowledge and human effort are required to develop ontologies. The yielding acceptable accuracy in plaintext clinical report classification with ontology-based ATC technique is a significant bottleneck [16–18]. Moreover, ontologies usually evolve because of constant changes in domain terminologies, thus, ontologies require manual effort to reflect changing vocabulary [17]. Therefore, these limitations show that ontology-based ATC technique is inappropriate for medical domain because of its high maintenance and manual effort.

Supervised ATC technique can be used to overcome challenges associated with ontology-based ATC technique. In supervised ATC, the domain expert first assigns a category or a class to each plaintext clinical report to create a training set. For instance, in case of an autopsy report, the pathologist assigns a cause of death to each report based on autopsy findings, such as “multiple injury” or “acute myocardial infarction,” after performing an autopsy examination. Second, all autopsy reports in the training set are tokenized into unique words to form a master feature vector. Third, various text classifiers can be applied on the master feature vector to build a decision model by classifying named entities from the source data being analyzed. Finally, the constructed decision model can be used in the new autopsy to assist pathologists in automatically determining the cause of death. The constructed decision model may accurately or inaccurately determine the cause of death because of numerous reasons [19]. Of these reasons, the most important is the selection of the most discriminative features in the decision model that correlate well with a specific cause of death. Thus, picking the finest features with highly discriminative power among various classes or causes of death is a complicated task and involves substantial effort in constructing a decision model [19]. Hence, this paper aims to develop an automatic multi-class classification system for predicting accident-related causes of death from free text autopsy reports by using expert-driven feature selection.

In this study, expert-driven feature selection approach, along with supervised ATC techniques, is used to achieve high-performance prediction of nine different accident-related causes of death from free medical text autopsy reports. Five different ATC classifiers were compared with the automated and expert-driven feature selection approaches to evaluate classifier performance by using macro precision (Precision_M_), macro recall (Recall_M_), macro F-measure (F-measure_M)_, and accuracy. This paper also investigates the effect of feature reduction on the overall performance of the decision model. In addition, the proposed expert-driven feature selection approach is compared with four baselines to show its significance. To the best of our knowledge, this paper is the first to use complete medical autopsy reports in determining accident-related causes of death.

This paper is organized in the following manner. In Section 2, the related work is presented. In Section 3, the methodology of this work is described. This section also includes the experimental setup and proposed feature selection approach. Section 4 presents the findings of the experiments. In Section 5, the findings are discussed. Section 6 also shows the significance of dataset and proposed feature selection approach. In addition, the proposed approach is compared with four baselines. Section 7 presents some limitations and future work. Finally, Section 8 concludes this work.

## 2.0 Related work

The supervised ATC techniques have been widely employed in the biomedical domain [20–28]. For instance, authors in [21] used support vector machine (SVM) text classifier to identify cancer-related causes of death from death certificates. The authors extracted features using term-based and concept-based features and used them to discover the discriminative features using information gain. Moreover, authors employed SVM and rule-based approach to classify death certificates they obtained for diabetes, influenza, pneumonia, and HIV diseases using term-based and concept-based features. Researchers in [29] developed a freely available graphic tool for biomedical text classification using various machine learning and text classification techniques. In [30], authors constructed and evaluated classifiers by employing SVM and Naive Bayes (NB)-supervised ATC techniques to classify the pathology reports.

In [31], researchers investigated the applicability and suitability of automatic text classification in epidemiological studies. Authors performed a comparative evaluation of a concept recognition approach and a variety of supervised ATC techniques, and they achieved 96.7% accuracy in the results. Authors in [32] explored the feasibility of using supervised ATC techniques in classifying clinical reports using NB and SVM text classifier, and they achieved 95% accuracy. The studies mentioned above gave us a concrete proof that the use of supervised ATC technique is appropriate for biomedical text documents, such as pathological reports, epidemiology reports, cancer-related reports, etc. However, very few studies employing supervised ATC techniques in predicting the cause of death from autopsy reports are available. This lack in related studies motivated us to contribute in this domain.

Perhaps, the work most related to our proposed work is that of [14]. In this study, authors used case-based reasoning approach coupled with the NB classifier and feature weight learning technique to support decision-making in forensic autopsy reports to determine the cause of death. Experimental results revealed that the CBR method, along with the implementation of a NB classifier, is a feasible approach of predicting the cause of death from forensic autopsy reports. Another important related work is that of [33], in which the authors used existing supervised ATC techniques to determine the cause of death using verbal autopsy reports. Here, authors used different combinations of linguistic and statistical features, such as unigram, bigram, and parts of speech tagging to extract useful features from verbal autopsy reports. Authors used SVM text classifier with various feature representation schemes to assist coroners and medical pathologist in determining the cause of death from the collected verbal autopsy reports, and they achieved 82.8% F-measure in predicting the time of death and 58.7% F-measure in the cause of death. However, the study only considered verbal autopsy reports in determining the cause of death.

On the other hand, both of these studies had two major limitations. The dataset used for cause of death prediction lack features mainly because the dataset only contains a brief summary or history-related information on the entire autopsy report. Authors did not consider other aspects, such as internal examination reports, external examination of the case, injury-related information, and other possible information in this study. However, history-related features are considered insufficient and are not discriminative to analyze the dynamics of an autopsy report. Therefore, considering further features from autopsy findings, such as injury-related findings, external examination findings, and internal examination findings can drastically improve the performance of prediction. The other limitation is the quality of features that depend on the source data being analyzed. Many pathologists may use different terms, synonyms, and vocabulary while preparing the autopsy reports. Hence, the consideration of various similar words using expert knowledge can further increase the accuracy of prediction. Therefore, an accurate, robust, and an efficient system must be developed to predict the cause of death by considering history-related features, internal examination-related features, and external examination-related features from free text autopsy reports. Moreover, an efficient and accurate technique for feature selection is needed to enhance the performance of a classifier.

## 3.0 Materials and methods

### 3.1 Data collection

The experiments involved 2200 autopsy reports on nine different leading causes of death related to accidents in Kuala Lumpur, Malaysia. These autopsy reports were collected from Rasmi Pusat Perubatan Universiti Malaya (PPUM) Hospital, Kuala Lumpur, Malaysia. The ethical letter provided by PPUM has also been attached in supplementary files. The detailed distribution of all nine classes is shown in Table 1. These reports were collected from one of the largest hospital of Kuala Lumpur, Malaysia. The causes of death on these reports were manually labeled unanimously by a team of pathologists. Each report consisted of the detailed examination of the dead body, including the deceased’s personal information, external examination, injury-related information, internal examination, history-related information, and information on histopathology reports. In the subsequent paragraph, the details of all these attributes are discussed. In addition, a sample of one autopsy report is also shown in S2 Appendix (please refer supporting information).

- Personal information: This section includes the name of the deceased, unique identity number, gender, date of birth, date of death, age upon death, and nationality.
- External examination: This part includes the information about the deceased’s external body parts, such as height, weight, eyes, ear, hands, feet, legs, nose, mouth, lips, teeth, and reproductive organs. Furthermore, information about rigor mortis, hypostasis, and decomposition signs is also recorded here. In addition, any specific symbols or patterns on the body are noted.
- Injury related information: This portion includes injury related information, such as the size, location, and pattern of abrasion, laceration, and wound on the body.
- Internal examination: This segment includes the anatomical examination of the brain, neck, thorax, cardiovascular system, respiratory system, gastro-intestinal tract, liver, spleen, pancreas, endocrine system, kidneys, and urinary bladder.
- Histopathology reports: This section includes the result of histopathology reports.
- History: This section records the previous history of the deceases and history of the day of death.
- Cause of death: This portion is the output variable of the autopsy report. Here, the experts process the autopsy findings, correlate the findings with previous cases, use their experience, and finally, decide the primary cause of death according to ICD-10 classification.

**Table 1:** The distribution of dataset across all nine classes.

S. No.	Cause of Death	ICD-10 Code	No. of Records	Gender	Age in years	Nationality	Total Distribution
1	Multiple Injury	T07	260	Male: 87% Female: 13%	Minimum: 14 Maximum: 87 Average: 39	Malay: 24% Chinese: 19% Indonesian: 7% Indian:24% Pakistani: 5% Bangladeshi: 19% Philippines: 2%	11.8%
2	Craniocerebral Injury	S06	260	Male: 84% Female: 16%	Minimum: 6 Maximum: 86 Average: 41	Malay: 31% Chinese: 35% Indian: 24% Indonesian: 6% Bangladeshi: 2% Philippines: 2%	11.8%
3	Abdominal Injury	S38	260	Male: 92% Female: 8%	Minimum: 20 Maximum: 50 Average: 30	Malay: 42% Indian: 15% Indonesian: 29% Pakistani: 14%	11.8%
4	Neck Injury	S17	260	Male: 80% Female: 20%	Minimum: 15 Maximum: 50 Average: 30	Malay: 40% Indian: 40% Pakistani: 20%	11.8%
5	Chest Injury	S28	250	Male: 89% Female: 11%	Minimum: 23 Maximum: 50 Average: 31	Malay: 34% Indian: 16% Indonesian: 34% Pakistani: 16%	11.4%
6	Liver Rupture	S36	250	Male: 67% Female: 33%	Minimum: 20 Maximum: 55 Average: 39	Malay: 33% Chinese: 33% Indian: 17% Pakistani: 17%	11.4%
7	Asphyxiation	T71	220	Male: 86% Female: 14%	Minimum: 5 Maximum: 50 Average: 24	Malay: 34% Chinese: 17% Indian: 16% Indonesian: 17% Pakistani: 16%	10.0%
8	Electrocution	T75	220	Male: 88% Female: 12%	Minimum: 5 Maximum: 44 Average: 24	Malay: 38% Chinese: 37% Indonesian: 25%	10.0%
9	Epileptic Seizure	G40	220	Male: 81% Female: 19%	Minimum: 17 Maximum: 50 Average: 33	Malay: 17% Chinese: 33% Indian: 17% Indonesian: 33%	10.0%

In experiments, personal information was not used in the prediction of cause of death because these features do not contribute to the prediction of the cause of death. Furthermore, all other features, such as external examination, internal examination, history, and injury-related features were concatenated in one string for the sake of simplicity. Next, all the reports were tokenized into words.

### 3.2 Master feature vector creation

A program was coded in Python version 3.4.3 using NLTK [34] to parse and to pre-process each plaintext autopsy report to determine the distinctive tokens present in all the autopsy cases. In pre-processing, four basic steps were performed. First, spell checker was used to correct all the misspelled words using PyEnchant and the NLTK library [34]. Second, the whole report was broken down into sentences after converting it into lower case, and each sentence was tokenized into words or tokens to form unigrams. The less common tokens which appeared only once or twice in the reports were also discarded because of their low occurrences. In addition, stop words were removed from the stop word list [35]. Third, each identified unique token was also represented by lexical categories using parts of speech tagger (POS-tagger) to identify the semantics of the token. For instance, after applying the POS tagger, the token or word “knee” was converted to “knee/Noun.” Finally, the tokens with their lexical categories were stored and represented in the master feature vector. This master feature vector was used for classification modeling by employing various supervised ATC techniques.

### 3.3 Feature engineering

To extract the highly discriminative features from the master feature vector, an expert-driven feature selection approach was used. Moreover, five automated feature selection techniques were also used to compare the performance of the proposed expert-driven feature selection approach.

#### 3.3.1 Expert-driven feature selection

**Fig 1:**
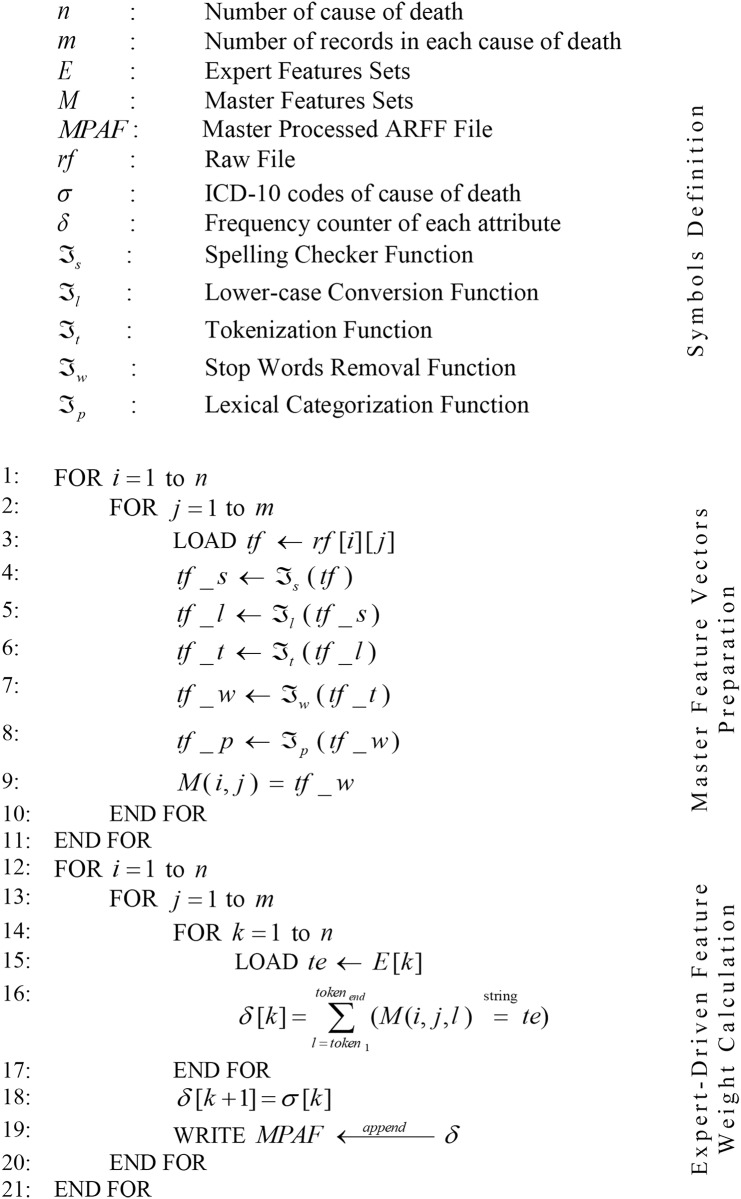
Algorithm of expert-driven feature selection approach.

The algorithm for the proposed expert-driven feature selection approach is given in Fig 1. Suppose we want to classify *n* different number of causes of death having unique *σ* ICD-10 cause of death code. Each cause of death comprises of *m* number of autopsy reports which are available in the *rf* raw files. For each cause of death, one expert feature set, *E*, exists. *E* contains the most discriminative features with ranked order list across all *n*. This *E* was prepared independently by two experienced domain experts. Moreover, in *E*, the possible synonyms and alternative words for the selected features were also added. Furthermore, both domain experts created the prioritized list of features that would predict the accurate cause of death from medical autopsy reports. Afterward, the domain experts matched their feature ranking and resolved their conflicts. A third pathologist was consulted to resolve the conflicts in case of disagreements. In this manner, *n* number of *E* was created.

Once we have the *n* number of *rf*, *m* number of autopsy reports in each *rf*, and *n* number of *E*, we then created the *n* number of *M* master feature vectors. To create the *M*, we first loaded one *rf* into memory and performed five different pre-processing tasks on each *m* in the *rf* to extract useful features from it. First, *ℑ*_*s*_ function was applied on *m* in *rf* to correct the misspelled words. Second, *ℑ*_*l*_ function was applied on *m* in *rf* to convert all words into lower case. Third, *ℑ*_*t*_ function was applied on each *m* in the *rf* to tokenize the autopsy reports into unique tokens. Fourth, *ℑ*_*w*_ function was applied on *m* in the *rf* to remove the most common words which do not contribute in the classification task. Finally, *ℑ*_*p*_ function was applied on each *m* in the *rf* to assign a lexical category or parts of speech tagging to each token. Finally, the processed *m* in the *rf* was stored in *M*. As such, all *n* number of *rf* were converted into *n* number of *M* that contained processed *m* autopsy reports.

After the creation of *M* and the preparation of *E*, *M* and *E* were loaded into memory. Eq 1 was applied on *M* and *E* to further process the *M* and form an ARFF file for classification. As shown, Eq 1 matches the tokens of *m* of *M* with each *E* and maintains the frequency count of the features of each *E* matched with the feature of *m* of *M*. Afterward, a unique ICD-10 cause of death (*σ*) was assigned to *m* of *M*, and this *m* of *M* was added to the ARFF file to create the training set.

$$ExpertDrivenFeatureWeight=\sum_{l=token_{1}}^{token{\;}_{end}}\left(M\left(i,j,l\right)\overset{string}{==}te\right)$$ (1)

#### 3.3.2 Automated feature selection

To compare the performance of proposed expert-driven feature selection approach, five automated feature selection schemes were also used were used to rank the most discriminative features from the master feature vector. These five automated feature selection techniques are: Chi-square [36], information gain [36], Pearson Correlation [36], Fisher Markov Selector [37], and improved global feature selection [38]. In automated feature selection techniques, the expert guidance was eliminated. Conversely, all tokens were ranked across all autopsy reports using these five automated feature selection schemes. Moreover, in all five automated feature selection schemes, subsets of highly discriminative tokens with maximum score were used as features for classifying the cause of death.

**(1) Information Gain:** Information gain, which identifies the importance of a given attribute in a feature vector, is the measure of reduction in uncertainty once the value of an uncertainty is known. If the uncertainty is largely reduced, knowing the value of an attribute provides a lot of information, and thus, we have a large information gain [8,15]. We assumed that the autopsy data set, D, have two classes, i.e., head injury (H) and multiple injury (M), with a total number of reports, d, containing h and m reports belonging to classes H and M, respectively. The amount of information is defined as shown in Eq 2.

I=−(h/d)log(h/d)−(m/d)log(m/d) (2)

If *h = m*, then *I* is equal to *1*, and if *h = s*, then *I* is equal to *0*. The information gain for autopsy dataset D using attribute A is then defined as shown in Eq 3.

$$Gain\left(D,A\right)=I-\sum_{i\varepsilon values\left(A\right)}\left(\frac{t_{i}}{S}\right)I_{i}$$ (3)

*I* is the number of information before split and $\sum_{i\in values(A)}\left(\frac{t_{i}}{s}\right)I_{i}$ is the sum of information after the split, where *I*_*i*_ is the information node *I*, and *t*_*i*_ is the number of objects in node *i*. Once the information gain was computed for every remaining attribute, the attribute with highest information gain was selected.

**(2) Chi-Square:** The Chi-square test is a statistical test that can be used in determining whether the observed frequencies of the tokens are significantly different from their expected frequencies [8,15]. For instance, consider the two classes, head injury (H) and multiple injury (M), in our autopsy dataset D having a total number of reports of 100. Out of the 100 reports, 50 belonged to class H, and the remaining 50 belonged to class M. Suppose both the classes contained the word “skull.” Therefore, the expected value for skull would be 50% for class H and 50% for class M, and the observed value would be the number of times the word “skull” appeared in class H and M. Therefore, Chi-square was defined as shown in Eq 4. Here, *O*_*i*_ refers to the observed or collected data, and *E*_*i*_ refers to the expected values.

$$x^{2}=\sum \frac{{\left(O_{i}-E_{i}\right)}^{2}}{E_{i}}$$ (4)

**(3) Pearson Correlation:** Pearson correlation measures correlation between two variables. Eq 5 shows the mathematical representation of Pearson Correlation. The value of *r* is between -1 to +1, where +1 represents high correlation and -1 represents negative correlation [36,39].

$$r\left(X,Y\right)=\frac{\sum_{i=1}^{n}\left(X_{i}-\bar{X}\right)\left(Y_{i}-\bar{Y}\right)}{\sqrt{\sum_{i=1}^{n}\left(X_{i}-\bar{X}\right)}\sqrt{\sum_{i=1}^{n}\left(Y_{i}-\bar{Y}\right)}}$$ (5)

**(4) Fisher Markov Selector (FMS):** Fisher Markov Selector (FMS) is an automated feature selection scheme that was proposed in [37]. FMS globally selects the optimal subset of features among the classes. This method is useful for handling high-dimensional data efficiently. In our experimental setup, we use FMS with linear polynomial kernel with *d = 1*, where *d* denotes the degree parameter [37].

**(5) Improved Global Feature Selection (IGFS):** The improved global feature selection (IGFS) scheme is an ensemble method where the power of global feature selection method and a one-sided local feature selection are combined in a different manner [38]. In our experimental setup, we have combined Odds Ratio as a one-sided local feature selection schemes and information gain as a global feature selection scheme [38].

### 3.4 Feature subset size

We hypothesized that various subsets of features would produce different performance results in terms of Precision_M_, Recall_M_, F-measure_M_, and overall accuracy. To evaluate this proposition, we selected feature subset sizes of 10, 20, 30, 40, and “all” after performing the sensitivity analysis (discussed in section 6). In addition, these subsets were extracted because of their implementation feasibility, thereby allowing the evaluation of classifier performance within a suitable operating range.

### 3.5 Text classification techniques

The features dug out from medical autopsy reports were used to build a decision model for accurately predicting the cause of death. Several machine learning classifiers were tested to select the best classifier. We tested SVM, NB, k-nearest neighbor (KNN), decision tree (DT), and random forest (RF) using Weka tool kit [8,40]. Five different text classifiers were employed because each classifier has a different philosophy behind the learning process. Moreover, these five classifiers have successfully been employed in text classification literature in the past. In subsequent paragraphs, these classification techniques are discussed.

#### 3.5.1 Naive Bayes (NB)

NB is one of the popular inductive learning classifier in supervised machine learning classifiers and is considered an efficient and effective decision model. The classifier has been widely employed in the classification of free text clinical reports [13,14,41,42]. NB is derived from Bayes' theorem with strong independence assumptions among features [43]. NB is very simple to use, fast, and it often produces better accuracy compared with other classifiers. Given a class variable C and dependent features f_1_ through f_n_, Bayes' theorem states their relationship as shown in Eq 6.

$$p\left(c|f_{1},f_{2},\ldots f_{n}\right)=\frac{p\left(c\right)p\left(c|f_{1},f_{2},\ldots f_{n}|c\right)}{p\left(f_{1},f_{2},\ldots f_{n}\right)}$$ (6)

Using the naive independence assumption, we obtained Eq 7.

$$p\left(f_{i}|c,f_{1},\ldots f_{i-1},f_{i}+1,\ldots f_{n}\right)=p\left(f_{i}|c\right)$$ (7)

For all i, this relationship is simplified as shown in Eq 8.

$$p\left(c|\left(f_{1},f_{2},\ldots f_{n}\right)\right)=\frac{p\left(c\right)\prod_{i=1}^{n}p\left(f_{i}|y\right)}{p\left(f_{1},f_{2},\ldots f_{n}\right)}$$ (8)

This technique is further discussed in [43].

#### 3.5.2 Support Vector Machines (SVM)

SVM is the popular supervised machine learning classifier and is based on statistical learning theories [44]. SVM has been proved to be an accurate classifier in many application areas such as image classification [45], and classification of biomedical documents [17,21,46]. SVMs are hyperplanes that separate the training examples by maximal margin [14]. Suppose we have two types of accident-related cause of death reports, i.e., liver rupture (L) and abdominal injury (A) in our autopsy reports. Given a training data of these autopsy reports (x_1_, x_2_,…x_n_) which are expressed in the master feature vector in a certain space X ≤. Rd. These instances are labeled as (c_1_,….c_m_), where c_i_ ε (L,A). Class L is on one side of the hyperplane, and class A is on the other side.

#### 3.5.3 K-Nearest Neighbor (KNN)

KNN employs instance-based learning. KNN is also termed as lazy learning classifier because it is the simplest classification algorithm that stores all the instances and classifies new instance using a similarity measure, such as the Euclidean distance shown in Eq 9 [47,48].

$$\sqrt{\sum_{i=1}^{k}{\left(x_{i}-y_{i}\right)}^{2}}$$ (9)

#### 3.5.4 Decision Tree (DT)

J48 is a popular DT classifier, and implementation of C4.5 decision model is used to create pruned or unpruned decision trees [49]. DT is the most commonly used algorithm for the task of classification and prediction [50]. The DT represents rules that can be easily understand by humans and constructs the classifier in hierarchical form. J48 classifier uses entropy to compute for the homogeneity of an autopsy report. J48 is discussed in detail in [49,51].

#### 3.5.5 Random Forest (RF)

RF is an ensemble supervised machine learning classifier that constructs multitudes of decision trees from training data using randomly selected features [52]. A new instance can be classified by all decision trees in a forest, and finally, the forest is responsible for choosing the classification decision using majority vote or by averaging the prediction using Eq 10.

$$f=\frac{1}{B}\sum_{b=1}^{B}f_{b}\left(x\right)$$ (10)

RF shows significant performance over a single DT. The classifier also overcomes the issue of overfitting. The major issue with RF classifier is its complexity, which minimizes interpretability and slows it down. RF was chosen as it was the best performing classification model in a previous text classification [53] and biomedical studies [54–57]. Moreover, M Fernández-Delgado, et al [58] compared 179 classifiers on 121 different datasets and they found that the RF is the best classifiers compared to other classifiers used in the study [58].

### 3.6 Experiments

**Fig 2:**
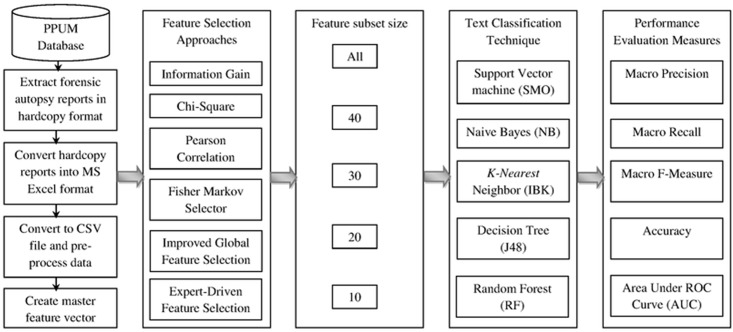
The complete flow of this research study.

The complete flow of this research study is shown in Fig 2. By utilizing six feature selection schemes (five automated feature selection schemes and one proposed expert-driven feature selection scheme), five subsets of features (10, 20, 30, 40, and “all” features), thirty (6 × 5) various feature sets were extracted for the construction and evaluation of text classifiers. Five different text classification techniques (NB, SVM, KNN, DT, and RF) were applied on each of these 30 feature sets with a total of 150 (5 × 30) analyses. All experiments were performed using 10-fold cross validation [59,60].

### 3.7 Evaluation metrics

In all 150 analyses, results obtained by each classifier with feature selection scheme and feature subset were compared. For performance evaluation, Precision_M_, Recall_M_, F-measure_M_, and overall accuracy were used. These performance measures were used because of imbalanced class distribution, and these metrics permit equal weights for each cause of death category [61]. In addition, receiver operating characteristics (ROC) curve and the area under the ROC curve (AUC) were also used to compare the performance of each cause of death category because of class imbalance [8]. In subsequent paragraphs, these evaluation metrics are discussed briefly.

#### 3.7.1 Macro precision (Precision_M_)

Precision_M_ is the average of each class precision; whereby the precision is the probability of test correctly predicted as positive cases given that the number of cases labeled by the system was positive. The mathematical definition of precision_M_ is shown in Eq 11.

$$Precision_{M}=\frac{\sum_{i=1}^{C}\frac{TP_{i}}{TP_{i}+FP_{i}}}{C}$$ (11)

#### 3.7.2 Macro recall (Recall_M_)

Recall_M_ is the average of each class recall; whereby the recall is the probability of the test finding the positive cases among all the cases of a given class. Recall is also known as sensitivity. The mathematical definition of recall_M_ is given in Eq 12.

$$Recall_{M}=\frac{\sum_{i=1}^{C}\frac{TP_{i}}{TP_{i}+FN_{i}}}{C}$$ (12)

#### 3.7.3 Macro F-measure (F-measure_M_)

F-measure_M_ is the weighted combination of precision_M_ and recall_M_. Mathematical definition of F-measure_M_ is given in Eq 14.

$$F-measure_{M}=\frac{\left(\beta^{2}+1\right)\text{Re}call_{M}\times \text{Pr}ecision_{M}}{\beta^{2}\left(\text{Re}call_{M}+\text{Pr}ecision_{M}\right)}$$ (13)

#### 3.7.4 Overall accuracy

Overall accuracy is the fraction of classification results predicted correctly among all the classes. Mathematical definition of overall accuracy is shown in Eq 14.

$$Accuracy_{Avg}=\frac{\sum_{i=1}^{C}\frac{TP_{i}+TN_{i}}{TP_{i}+FN_{i}+TN_{i}+FP_{i}}}{C}$$ (14)

#### 3.7.5 Area Under ROC Curve (AUC)

The area under ROC curve or simply AUC has been recently introduced to evaluate machine learning algorithms [62,63]. This measure is very useful in analyzing the performance of the decision model with respect to a specific class. The AUC provides a good summary for the performance of the ROC curves. The ROC is a method to calculate the goodness of machine learning classifier by plotting a specific curve and calculating the area under this curve. It is instinctively obvious that for good performance algorithm the value of AUC will be close to 1 and the value of 0.5 or less than that indicates the poor performance of algorithm [63–65]. Hand and Till [64] presented a simple method to calculate the AUC of decision model using Eq 15. Here, *n*_0_ and *n*_1_ represent the number of positive and negative examples respectively, and $S_{0}=\sum r_{i}$, where *r*_*i*_ is the rank of *i*_*th*_ positive example in the ranked list. The detail of AUC has been discussed in [63–65].

$$AUC=\frac{S_{0}-n_{0}\left(n_{0}+1\right)/2}{n_{0}n_{1}}$$ (15)

## 4.0 Results

From all 2200 autopsy reports, after applying the pre-processing steps, a total of 19164 unique tokens were identified. From the 19164 tokens, 9543 tokens were removed because they appeared once or twice in the whole autopsy reports set. The remaining tokens were stored in the master feature vector and evaluated using the aforementioned six feature selection approaches with subsets of 10, 20, 30, 40, and “all” tokens using five classifiers (please refer S1 Appendix in supporting information). Results of each of the 150 analyses in terms of accuracy, Precision_M_, Recall_M_, and F-measure_M_ are as follows. Moreover, the AUC graphs of the best performing technique which produced the highest accuracy and highest F-Measure_M_ are also shown in the results.

**Fig 3:**
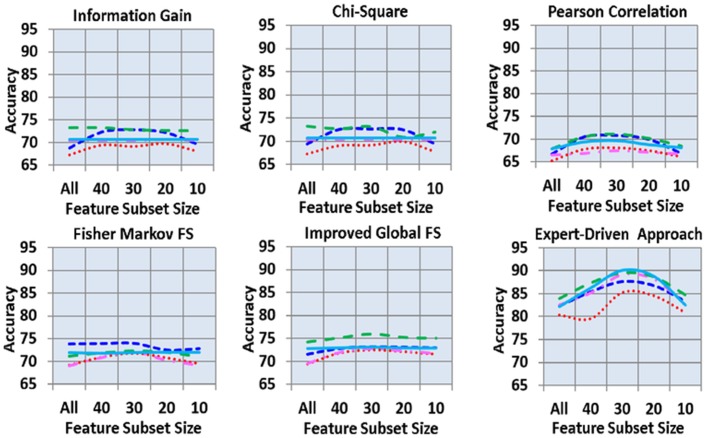
Overall accuracy across feature selection schemes, subsets of features and classifier.

Fig 3 shows the overall accuracy of all 150 analyses. In the figure, the expert-driven feature selection scheme significantly outperformed all five automated feature selection schemes, followed by IGFS scheme and FMS scheme. In addition, information gain and Chi-square produced the almost same results. The lowest results were shown by Pearson Correlation scheme. A fluctuating trend was found in the feature subset size. However, the lowest accuracy was observed in the “all” and 10 feature subset sizes. The reasonable accuracy was found in the feature sub set sizes of 20, 30, and 40, respectively. J48 classifier outperformed in all automated feature selection schemes excluding FMS by producing the highest accuracy of 75.94% (with a feature subset size of 30), followed by SVM (73.15%, with feature subset size 30) and RF (73.05%, with feature subset size 40). Moreover, the lowest performance was observed in NB classifier which produced 67.31% accuracy in the “all” feature subset size, followed by KNN (70.36%, with feature subset sizes of 20 and 10). In FMS feature selection scheme, SVM outperformed all other classifiers. Conversely, RF and J48 classifiers outperformed in expert-driven feature selection scheme by producing the highest accuracies of 90.09% and 89.50%, respectively, using a feature subset size of 30. In addition, KNN also showed an accuracy less than that of J48. The lowest accuracies of 80.31% and 82.31% with feature subset size of “all” was found in NB and SVM, respectively.

**Fig 4:**
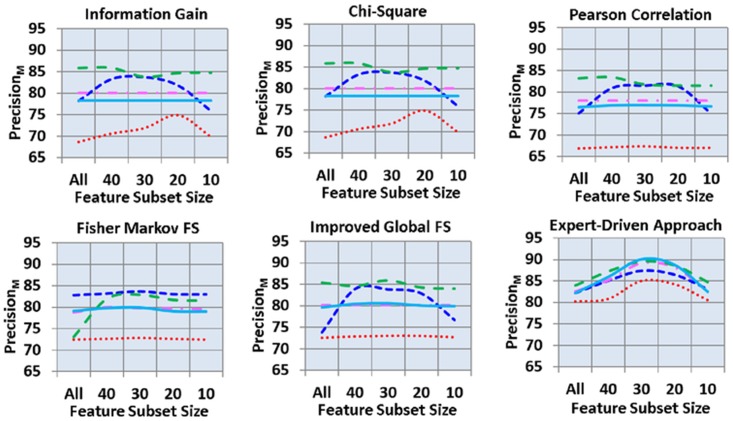
Macro precision across feature selection schemes, subsets of features and classifier.

Fig 4 shows the precision_M_ of all 150 analyses. As shown here, in the automated feature selection schemes, IGFS produced the highest precision_M_, followed by FMS. Information gain and Chi-square schemes yielded roughly the same results and the lowest precision_M_ was observed in Pearson correlation. Furthermore, J48, SVM, and RF classifiers produced the highest precision_M_ of 85.93%, 83.83%, and 80.65% respectively, with the feature subset size of 30. In addition, NB and RF produced the lowest precision_M_ of 68.71% and 78.20%, respectively, with the feature subset of “all.” However, in expert-driven feature selection scheme, RF, J48, and KNN produced the highest precision_M_ of 90.10%, 89.50%, and 89.10%, respectively, using the feature subset size of 30. Moreover, SVM and NB yielded the lowest precision_M_ of 82.20% and 80.20%, respectively, with the feature subset size of “all.”

**Fig 5:**
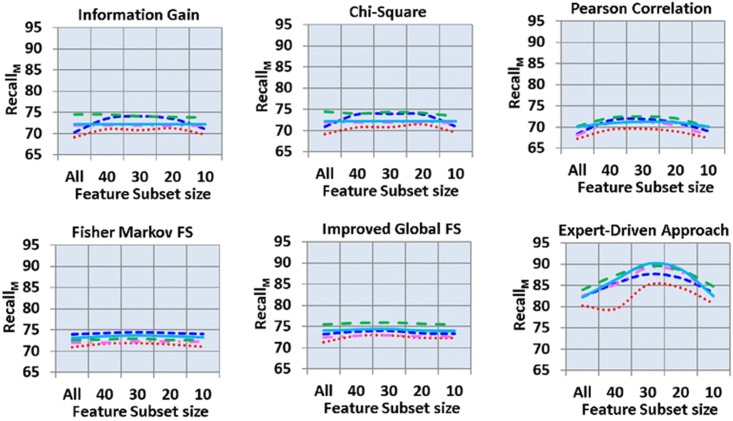
Macro recall across feature selection schemes, subsets of features and classifier.

Fig 5 shows the recall_M_ of all 150 analyses. The figure shows that the expert-driven feature selection scheme outperformed the automated feature selection schemes. In automated feature selection schemes, the highest recall_M_ was observed in IGFS scheme. Moreover, the Minor difference was observed with the results produced by FMS, information gain and Chi-square automated feature selections schemes. The lowest recall_M_ was observed in Pearson Correlation. Majority of the developed models yielded the lowest recall_M_ with feature subset sizes of “all” and 10 and the highest recall_M_ with feature subset sizes of 20 and 30. In automated feature selection schemes, J48 decision model outperformed by yielding a 75.93% recall_M_ with a feature subset size of 30. Furthermore, the recall_M_ produced by RF with a feature subset size of 30 was slightly lower than the recall_M_ of J48. In addition, NB and KNN decision models showed the lowest recall_M_ of 69.10% and 71.81% with feature subset sizes of “all” and 40, respectively. However, in expert-driven feature selection scheme, RF, J48, and KNN produced the highest recall_M_ of 90.10%, 89.50%, and 89.10%, respectively, with feature subset size of 30. Moreover, SVM and NB showed the lowest recall_M_ of 82.30% and 80.30%, respectively, with a feature subset size of “all.”

**Fig 6:**
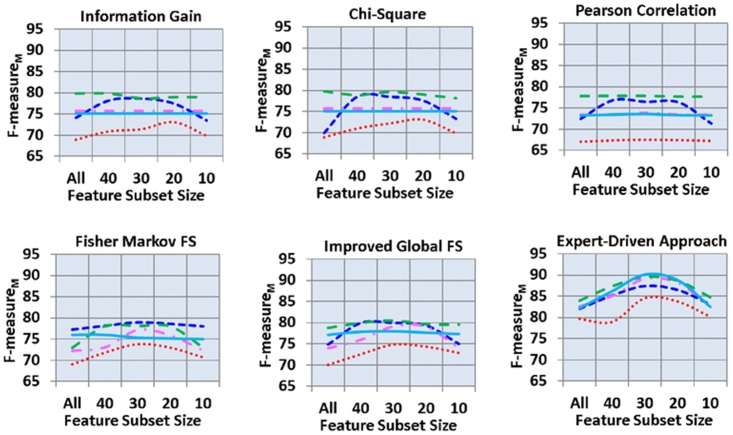
Macro F-measure across feature selection schemes, subsets of features and classifier.

Fig 6 shows the F-measure_M_ of all 150 analyses. Here, the highest F-measure_M_ was produced by the expert-driven feature selection approach. In automated feature selection schemes, IGFS scheme outperformed all other four automated feature selection schemes, followed by FMS scheme. Moreover, in many decision models, both information gain and Chi-square automated feature selection schemes produced similar results with extremely minute fluctuations. The Pearson correlation yielded the lowest F-measure_M_. Majority of the decision models yielded the lowest F-measure_M_ with feature subset sizes of “all” and 10 and highest F-measure_M_ with feature subset sizes of 20, 30, and 40. In automated feature selection schemes, J48 decision model produced the highest F-measure_M_ of 80.51%, with a feature subset size of 30 followed by SVM (79.94%, with feature subset size of 30). Moreover, the lowest F-measure_M_ of 68.80% was observed in NB decision model with the feature subset size of 10. However, in expert-driven feature selection scheme, RF, J48, and KNN showed the highest F-measure_M_ of 90.10%, 89.50%, and 89.10%, respectively, with a feature subset size of 30. Furthermore, SVM and NB showed the lowest F-measure_M_ of 82.10% and 79.90%, respectively, with feature subset size of “all.”

**Fig 7:**
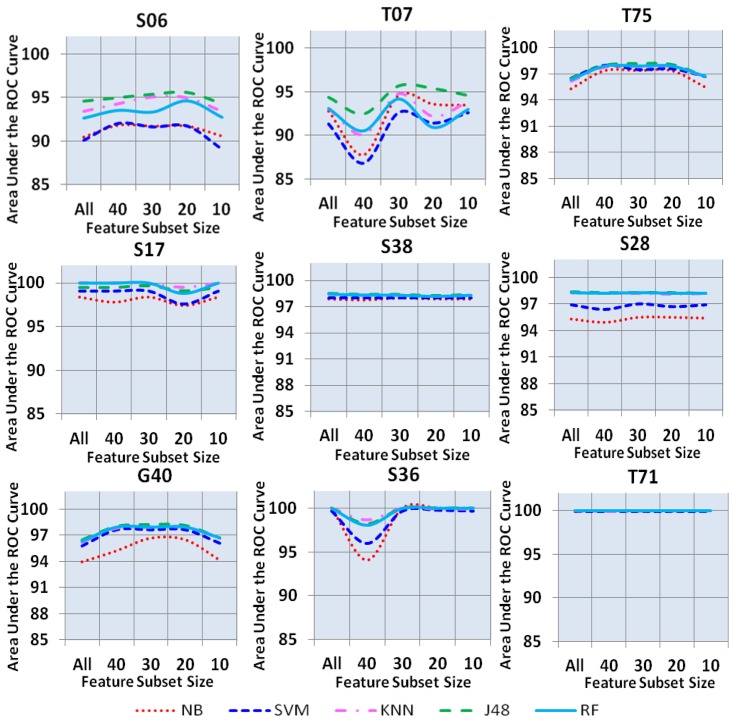
Area under the ROC curve for all nine classes.

In Fig 7, the AUC of all nine classes (T07, S06, S38, S17, S28, S36, T71, T75, and G40) using expert-driven feature selection approach is shown. Results revealed that in class “T71,” 100% AUC was achieved by all the five classifiers with all the five feature subset sizes. Moreover, in class “S36,” all five classifiers produced 100% AUC with all subset sizes excluding the feature subset of 40, whereas an irregular trend was observed in the AUC of all five classifiers. In class “S17,” RF, J48, and KNN yielded an almost 100% AUC with all the five feature subset sizes. However, in “S17,” the AUC observed in NB and SVM is higher than the AUC of RF, J48, and KNN. In class “S38,” AUC of 98% was observed in all five classifiers with all five feature subset sizes. Almost a similar type of irregular trend of AUC was found in classes “T75,” “G4,”' and “S28,” where all classifiers yielded an AUC in between 95%–98% with all the five feature subset sizes. The lowest AUC (87%–93%) was produced by NB and SVM in classes “S06” and “T07” with all five feature subset sizes. Nevertheless, RF, J48, and KNN achieved approximately 95% AUC in “S06” and “T07” with all the five feature subset sizes.

From Fig 7, the RF decision model parameterized with expert-driven feature selection approach using a feature subset size of 30 correctly classified approximately 95% of the “S06” and “T07” causes of death and misclassified 5% in other classes. Moreover, RF decision model parameterized with expert-driven feature selection approach using feature subset size of 30 correctly classified approximately 98% of “T75,” “S38,” “S28,” and “G40” causes of death and misclassified 2% in other classes. Furthermore, RF decision models parameterized with expert-driven feature selection approach using feature subset size of 30 correctly classified approximately 100% of “S17,” “S36,” and “T71” causes of death. Therefore, the RF parameterized with expert-driven feature selection approach using feature subset size of 30 can be concluded as a feasible solution for predicting ICD-10 causes of death in free text autopsy reports.

**Fig 8:**
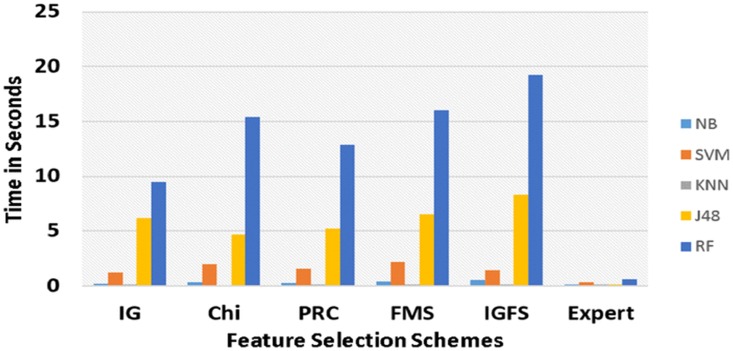
Computational time analysis of decision models and feature selection approaches.

In text classification task, one of the crucial performance measures is the computational time taken by classifier in building the classification model. Fig 8 shows the average computational time for all five classifiers in all five feature subset sizes by using six feature selection schemes. All 150 analyses were run on Corei7 system having 2.80 GHZ clock speed and a 16-gigabyte memory. As shown here, the proposed expert-driven approach is much faster than the automated feature selection schemes. Moreover, in automated feature selection, information gain proved to be faster than other four automated feature selection schemes. In all six aforementioned feature selection schemes, KNN and NB required the least time to construct the decision model. Nevertheless, in the majority of the experiments, J48 and RF showed the highest accuracy, precision_M_, Recall_M_, and F-measure_M_, however, they both took the longest computational time to build a decision model.

## 5.0 Discussion

The experimental results of this research study show that supervised ATC techniques can identify the cause of death from free text medical autopsy reports with performance measures between 70%–90%. Furthermore, a considerable difference was observed in most of the analyses. From the experimental findings, different combinations were determined to optimize the performance of each measurement.

To optimize the overall accuracy, precision_M_, and F-measure_M_, RF decision model built with expert-driven approach using a subset of 30 features is recommended. Experimental results also indicate that in automated feature selection approaches IGFS scheme sowed the highest performance, followed by FMS. Moreover, in most of the experiments, information gain produced results that are almost similar with those returned by Chi-square. Pearson Correlation showed the lowest performance results in all of the experiments. However, expert-driven feature selection approach produced much better results than all five automated feature selection approaches used. Two primary reasons might account for the better performance of expert driven feature selection approach. First, all the 2200 cases belonged to the group with “accident” manner of death. All nine different classes under consideration were very similar in nature. Furthermore, numerous tokens were common across all the autopsy reports. For instance, the tokens “abrasion”' and “laceration” are highly related to all types of accident under consideration. Second, different pathologists might have used different synonyms and vocabulary while preparing the autopsy reports. For instance, many pathologists used the tokens “abrasion,” “graze,” and “trauma” interchangeably. Therefore, we suggested to experts during the creation of expert-driven features to select the features that were the most discriminative to a particular cause of death. In addition, experts were also suggested to come up with a possible set of synonyms of selected features. Hence, the resultant expert-based feature space comprised of rich set of discriminative features for each cause of death under consideration. Therefore, potential researchers should not only rely on results produced by automated feature selection but should also explore more features with the help of domain experts. Moreover, the proposed expert-driven feature selection approach was much faster than all five automated feature selection approaches. Such result was caused by algorithms, such as IGFS, FMS, Chi-square, information gain and Pearson Correlation, in automated feature selection which consider the whole dataset in determining the most discriminative features by applying various computational methods.

However, in expert-driven feature selection approach, the features were already provided and ranked by experts, hence, this approach only calculated the expert-driven feature weighted from autopsy reports and prepared the classification data using frequency count. Furthermore, the classification file prepared by expert-driven feature selection approach was much smaller in size compared with that of five automated feature selection schemes. The classification file prepared by expert-driven approach only contained the number of attributes equivalent to the number of classes. Conversely, the other five automated feature selection schemes counted each token as one feature after tokenization, and the number of attributes was equal to the number of unique tokens. Therefore, the automated feature selection techniques required longer classification time. Finally, the proposed expert-driven feature selection can be used in classifying any kind of clinical reports. The only thing required by this approach is the ranked features from an expert.

The accuracy of classification task usually depends upon the quality of features set. The inadequate, extraneous, and irrelevant features may generate less accurate and incomprehensible results. Therefore, it is an important task to remove irrelevant and non-discriminative feature subset from master feature set by using feature subset selectors algorithms prior to classification [66]. The purpose of feature subset selection is to decide which number of features to include in classification and which to remove. For this research, we also hypothesized that various subsets of features would produce different performance results in terms of Precision_M_, Recall_M_, F-measure_M_, and overall accuracy. To evaluate this proposition, we aim to determine the best feature subset size for the classification of autopsy reports to improve the classification performance. To discover the best feature subset size, initially, the subset of 10 features were selected using all aforementioned six feature selection schemes to evaluate the performance of all five classifiers. The number of features were increased up to the point where no further improvement in performance was found. In addition, we also evaluated the performance of all five classifiers using ‘all’ features. In most of the experiments, we noticed that increasing the size of feature subset from 10 to 30 led to considerable improvements in experimental results. Conversely, increasing the size of feature subset from 30 to 40 to “all” did not cause considerable improvements in the results. As a result, we can infer that a feature subset of larger size may not positively affect the results. Thus, to determine an optimum size of features in feature vector, researchers are suggested to perform sensitivity analysis to examine a range of feature sizes from point 10 to a point where no improvement in accuracy is observed.

According to the “no free lunch” theorem [67], there is no single machine learning algorithm that performs best in all application areas. Hence, a variety of decision models should be tested. Therefore, we evaluated the performance of five classifiers (NB, SVM, kNN, J48 and RF) with six aforementioned feature selection approaches to classify free text autopsy reports. Here, in four automated feature selection approaches namely, information gain, chi-square, Pearson Correlation and IGFS, J48 classifier produced the most promising results, followed by SVM and RF. In FMS feature selection scheme SVM produced the highest results, followed by J48 and RF. Conversely, in expert-driven feature selection approach, RF outperformed the other classifiers, followed by J48. There may be various possible reasons for the outperformance of J48 in automated feature selection such as J48 does not need any domain knowledge or any parameter setting and it can handle data with high dimensionality. Moreover, J48 can handle datasets with errors and missing values. Furthermore, it is considered as a nonparametric classifier which means it does not use any assumptions for space distribution and classifier structure. The main disadvantage of J48 is that it can easily over fit. The possible reason for outperformance of RF has the best result because of its ensemble nature. RF constructs multitudes of decision trees from training data using randomly selected features. To classify a new autopsy reports from an input dataset, RF put the input vector down each of the trees in the forest. Each tree predicts the CoD for given autopsy report and finally the forest chooses the final CoD using having the most votes. SVM, KNN, and NB showed a considerable lower performance than RF and J48 in all the experiments. We speculated that NB supposes a conditional independence among features that is possibly inappropriate for the collected autopsy reports [68]. Furthermore, as the number of features increases, the conditional dependence among the features becomes more complicated, and this can negatively affect the performance of NB classifier. The reason behind the poor performance of KNN classifier may be its default supposition of linear scaling of features that might have led to the inaccurate computation of KNN distance measures. In addition, this assumption becomes misleading with features having very low discriminating power. The performance of SVM lies in the choice of kernel [69]. The selection of proper SVM kernel and kernel function parameters, such as width or sigma parameter, may further increase the SVM performance [70]. In our future optimization work, we might find the optimal parameter values for SVM decision model.

## 6.0 Significance of dataset and proposed feature sets

**Fig 9:**
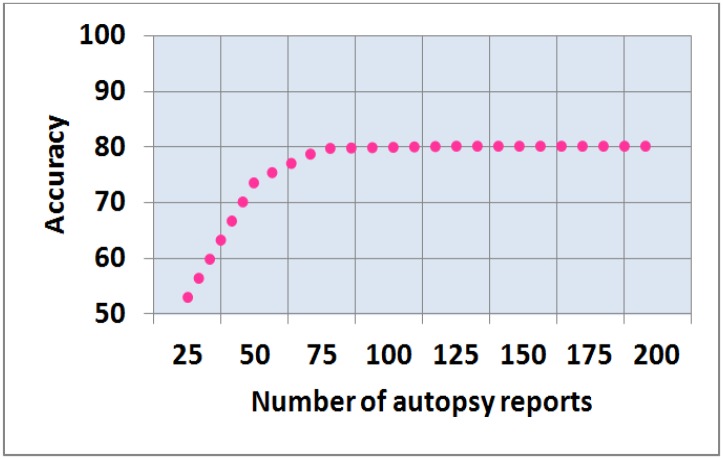
Decision model accuracy versus number of autopsy reports.

In medical autopsy, suitably annotated and statistically independent samples of autopsy reports for the construction and evaluation of classifier are inadequate and expensive. In addition, ethical considerations often restrict the number of autopsy reports collection. Thus, sample size planning is an important aspect in the design of experiments. Hence, to find the optimum sample size for each class, various experiments were performed to examine a range of sample size from 25 to a number of instances where no further improvement in accuracy was observed. Here, all the experiments were performed using expert-driven feature selection approach with feature subset size of “all” and RF classifier. The expert-driven feature selection approach with RF classifier was used in this study because it produced the best results in all the performed experiments. Results of these experiments are shown in Fig 9. Here, the lowest accuracy of 52%–73% was noted when the number of reports were 25 to 50. Accuracy between 79.80%–80.05% was observed with 75 to 100 autopsy reports. The extremely slight variation in accuracy was observed when the cases increased from 100 to 200. A consistent accuracy of 80% was found in 125 to 200 autopsy reports. Thus, we concluded that a minimum of 75 to 100 autopsy reports are a reasonable number for constructing and evaluating an accurate model for the classification of medical autopsy reports.

Given the restrictions brought about by privacy or ethical considerations, no public dataset was available for testing the significance of the proposed approach. To examine such significance, four baselines were created from the collected dataset for this research, namely, bag of words (BoW) and the combination of feature engineering techniques proposed in[14], [41] and [71,72]. In [14] rule based feature weight learning technique was used to select the features from forensic autopsy reports. In [41] locally-semi-supervised feature selection technique was used to select the most discriminative features from verbal autopsy reports belonging to each CoD. In [71,72] “Ensemble-based Multi-Filter Feature Selection (EMFFS)” was used to select the most important and the most discriminative features for the classifier by combining many filter based feature selection schemes. To compare, our proposed approach with EMFFS, four feature selection methods were combined, namely, chi-square, information gain, Pearson correlation and gain ratio using Weka tool. Finally, we combined the output of these four feature selection schemes using a fusion based rank aggregation method proposed in [73] to generate a final global features ranking list.

The experiments were conducted to measure the overall accuracy of all five classifiers using these four baseline features. The baseline accuracy was compared with the accuracy of the proposed expert-driven feature selection approach using the “all” feature subset size. The accuracy of baseline feature selection schemes and proposed expert-driven feature selection scheme is shown in Table 2. As shown in the table, in all four baselines, the J48 and RF decision models consistently showed a promising accuracy and the lowest accuracy was observed in KNN, NB and SVM. In all four baselines, the highest accuracy of 73.85% and 73.18% was obtained by J48 and RF in baseline 4. However, compared to all these baselines, our proposed expert-driven feature selection technique showed the promising results.

**Table 2:** Comparison of accuracy results of baselines approaches and proposed approach.

Classifier	Baseline 1	Baseline 2 [41]	Baseline 3 [14]	Baseline 4 [71,72]	Proposed Approach
NB	71.59	70.13	67.72	72.59	80.31
SVM	69.81	68.90	68.40	72.81	82.31
KNN	70.45	70.45	70.45	70.50	82.59
J48	72.45	72.68	73.18	73.85	83.90
RF	70.72	70.72	70.72	73.18	83.18

## 7.0 Limitations and future work

Some of the challenges were also identified in the proposed expert-driven feature selection approach. First, results of the proposed expert-driven approach depend heavily on the domain knowledge of the experts and their familiarity with autopsy findings. We believed that in the current study, the engagement of pathologists yielded experimental results that can be reflected across other medical systems. Second, the presented findings are exclusive to the free text autopsy reports obtained from PPUM, one of the largest hospital in Kuala Lumpur, Malaysia. We also believed that the quality of the extracted reports is sufficiently heterogeneous, diverse, and comprehensive compared with the data gathered by other medical systems and therefore should produce acceptable results across other healthcare systems. Third, the developed model can only detect nine accident-related causes of death. However, the system can be enhanced by following similar steps of detecting various accident-related causes of death. Finally, this paper proposes the use of supervised ATC techniques to predict the cause of death from autopsy reports. However, ontology-based approach may produce better results than our proposed approach. Meanwhile, because the proposed method resulted in performance measure exceeding 90%, slightly better results may not support the significant ontology development efforts.

Various opportunities were also identified for the improvement of a system that will require future work on the domain under consideration. First, reviewing autopsy reports and assigning the cause of death on each autopsy reports by pathologists for the preparation of training set for classification purpose is a time-consuming and challenging task. Therefore, we suggest evaluating the presented results produced by the proposed classification task against those of clustering techniques that require unlabeled data. Moreover, compared with the classification approach, clustering is painless to implement, and it requires less involvement of experts in system implementation. Second, though our proposed expert-driven feature selection approach produced a satisfactory performance, however, in future work, we aim to employ the ontology-based approach to compare its results with our findings. Finally, in future work, we aim to employ the supervised ATC and ontology-based techniques on autopsy reports to predict heart-related causes of death and homicide-related causes of death.

## 8.0 Conclusion

In this paper, an expert-driven feature selection approach was proposed to predict the cause of death from free text medical autopsy reports. Moreover, the state-of-the-art supervised ATC techniques with automated and expert-driven feature selection approaches were used to classify the cause of death from free text medical autopsy reports. We discovered that the proposed expert-driven feature selection approach outperformed in terms of performance measures exceeding 90% when compared with automated feature selection approaches. Moreover, RF and J48 classifier was found to be suitable for the classification of autopsy reports with a feature subset size of 30. Based on the results, the proposed system proved to be more robust and more accurate when it was compared with four baselines. Furthermore, the promising results indicate that the pathologists can use the proposed system as a source of second opinion, assisting them in more accurately and rapidly determining the cause of death. In addition, this research can be enhanced to assist other clinical reports. The proposed technique has the capability to cut down the time and effort needed for public healthcare reporting.

## Supporting information

S1 Appendix Top 30 automated and expert-driven features. (PDF)

S2 Appendix Sample autopsy report. (PDF)
