# Supplementary material for: Automatic ICD-10 multi-class classification of cause of death from plaintext autopsy reports through expert-driven feature selection
Source: PLoS One. 2017 Feb 6;12(2):e0170242. doi: 10.1371/journal.pone.0170242 (PMC5293233; doi:10.1371/journal.pone.0170242)
Supplement: S1 Appendix — (PDF) [file pone.0170242.s001.pdf]

S1 Appendix: Top 30 Automated and Expert-Driven Features

Automatic using Chi-Square	Automatic using Information Gain	T07	S06	S38
Deceased	Unremarkable	Acute	scalp	subconjuctival
Unremarkable	Deceased	subscalpal	temporal	symphysis
Showed	Showed	bruise	frontal	pubic
Left	Police	cervical	subarachnoid	renal
Police	Upper	thoracic	arachnoids	vessel
Upper	Abrasion	vertebrae	hemorrhage	adernal
Abrasion	Fracture	sacroiliac	leptomeninges	kidney
Fracture	Measuring	joint	extradual	parenchyma
Measuring	Lungs	shock	intracerebral	pale
Found	Surface	grazed	tentorial	wound
Surface	Back	ramous	herniation	paraumbilical
Back	Found	calcification	aneurysm	area
Lungs	Weighed	thrombosis	ventricles	nail
Weighed	Knee	embolism	venous sinuses	beds
Blood	Blood	stenosis	cranial	bluish
Multiple	Multiple	subendocardial	odema	discolouration
Knee	Chest	sternum	nerves	Abdominal
Body	Intact	perennial	cerebro	Wall
Aspect	Aspect	bladder	spinal	Blunt
Heart	Body	hemothoran	fluid	Penetrating
Intact	Brain	infarction	cerebrospinal	Trauma
Dead	Heart	fibrosis	cerebral	Obstruction
Medical	Pulmonary	pulmonary	vessels	Instestine
Chest	Medical	embolism	thrombosis	Rupture
Spinal	Dead	thrombo	preorbital	Distention
Brain	Spinal	oedema	mandible	Gastrointestinal
Aorta	Small	mural	sphenoid	Contusions

Automatic using Chi-Square	Automatic using Information Gain	T07	S06	S38
Sections	Skull	thrombi	circle	Thoracic
Lower	Scalp	limbs	Willis	Pain
Skull	Laceration	ulna	ruptured	Bike

S28	S17	S36	T71	G40
Ribs	hyoid	Peripheral	body	Alvealar
Fractures	bone	Cynosis	decomposed	Distension
Pnemothorax	fracture	Hematoma	gaseous	Anticonvulsant
Haemo-Pneumothorax	multiple	Liver	belbs	external
Haemopneumothorax	abrasion	Laceration	diaphram	surface
Pericardium	cervical	Lobe	respiratory	Focal
Guilletine	verteberal	Rupture	passages	Tongue
Spleen	neck	Patellar	reddish	Bite
Clavicle	muscle	Soft	black	Edema
Intercostal	contusion	tissue	Oxyden	Diffuse
Perforation	tracheal	Granular	honey comb	Sinusoidal
Inter-atrium	ring	Bilateral'	lung	Abnormal
Interatrium	thyroid	Cystic	oedematous	Cortex
septuma	gland	Acetabular	haemopericardium	Fit
Inter-ventricular	Acute	Trauma	chest	Disorder
Inteventricular	Death	Blunt	wall	Glaucoma
Septum	Upper	Anterior	hamatoma	Dementia
Haemorhages	Spinal	Vehicle	haemothorax	Epilepsy
Haemorrhages	Cord	Injury	frothy	Schizophrenia
Endocardium	Hyperextension	Abdomen	secretion	Cerebral
Lungs	Necrosis	Bruise	congested	Sclerosis
Alveoli	Larynx	Intact	thyroid cartilage	Mental
Glands	Trauma	Upper	upper	Illness
Kidneys	Back	hemoperitoneum	midsternum	Neurofibromatosis
Bike	Broken	hemopericardium	mid-sternum	Sleep

S28	S17	S36	T71	G40
Alimentary	pharynx	Tears	Oxygen	Apnea
Tract	Spine	Bleeding	Asphysia	Hemiplegia
Ventilation	Nerves	Blood	Unconscious	Migraine
Trauma	Intervertebral	Swollen	Breathing	Autism
Blunt	Tissue	Cavity	Convulsions	Unconscious

T75	
Face	Charring
Congestion	Burn
Collapsed	Marks
Blister	Shock
Parietal	Red
Lpeural	Voltage
Thickening	Breath
Aspirated	Electric
Gastric	Skin
Contents	Heels
Petechial	High
Electricity	Bones
Haemorrhage	Heart
Oedamatous	Numbness
Hand	Tingling
