# Supplementary material for: Automatic ICD-10 multi-class classification of cause of death from plaintext autopsy reports through expert-driven feature selection
Source: PLoS One. 2017 Feb 6;12(2):e0170242. doi: 10.1371/journal.pone.0170242 (PMC5293233; doi:10.1371/journal.pone.0170242)
Supplement: S2 Appendix — (PDF) [file pone.0170242.s002.pdf]

S2 Appendix: Sample Autopsy Report

Autopsy: BPD110094-67F

DECEDENT: John Anderson

Autopsy Authorized by: Dr. Smith for City of Indianapolis.
Identified by: fingerprints and dental comparison.

Rigor: absent
Livor: purple
Distribution: posterior
Age: 34
Race: White
Sex: Male
Length: 72 inches
Weight: 185 pounds
Eyes: Hazel
Hair: Brown
Circumcised: Yes
Body Heat: Refrigerated

CLOTHING:

1. Blue dress shirt, blood-stained and scorched. Multiple tears of different sizes on the front. Both lower arms are shredded.
2. White T-shirt, blood-stained and scorched with multiple tears of different sizes on the front matching the tears on the blue dress shirt.
3. Red tie shredded, scorched and blood-stained.
4. Brown pants, blood-stained with irregular tears, scorching, and soot deposition, primarily on the left upper leg and crotch.
5. Brown belt.
6. White underwear briefs, torn on left side with searing, soot deposition and blood stains.
7. Brown socks.
8. Black shoes.

EXTERNAL EXAMINATION:

Well developed, well nourished, white male with multiple blast and fragment injuries including amputations of both upper extremities at mid-forearm with partial fragmentation of the detached portions of the extremities. There are lacerations of face and scalp with fractures of the calvarium, skull base and facial bones. The cranial vault is empty with fragments of brain recovered separately.

There are multiple penetrating fragment injuries of the anterior thorax and left thigh. There are gaping lacerations of the left lower abdomen and thigh with partial evisceration of sigmoid colon and small intestine. The penis and left testicle are absent. There are flash burns of the anterior thorax, face and left thigh.

X-RAYS:

Total body x-rays demonstrate comminuted fractures of the face and skull, multiple anterior rib fractures, comminuted fractures of pelvis and proximal left femur, amputations of distal upper extremities, and multiple metallic fragments in the head, thorax, and thighs.

HISTORY:

Injured by exploding package.

PATHOLOGICAL DIAGNOSES:

1. Blast and fragment injuries to head
    a. Comminuted fractures of calvarium, skull base and facial bones
    b. Avulsion and fragmentation of brain
    c. Avulsion and fragmentation of eyes
    d. Penetrating fragment injuries to lower face
    e. Flash burns to lower face
2. Blast and fragment injuries to trunk
    a. Multiple perforation injuries with multiple lacerations of lungs, heart, liver, spleen, and intestines
    b. Multiple fractures and perforations of anterior ribs and sternum
    c. Laceration of left lower abdominal wall with partial evisceration of small intestine, mesentery, and colon
    d. Flash burns to anterior thorax
    e. Comminuted fractures of left ilium, ischium and pubic ramus with separations of the sacro-iliac joints and pubic symphysis.
    f. Multiple metal fragments recovered from thorax, abdomen and pelvis.
3. Blast and fragment injuries to extremities
    a. Amputation of right hand and forearm with avulsion and searing of muscle and connective tissue
    b. Amputation and fragmentation of left hand and forearm with avulsion and searing of muscle and connective tissue
    c. Multiple penetrating fragment injuries to anterior surface of right arm

d. Comminution of left proximal femur with partial avulsion and searing of muscle and soft tissue.
e. Multiple metal fragments recovered from soft tissues of left thigh

4. Toxicology
   a. Blood carboxyhemoglobin less than 5% saturation
   b. Blood and vitreous fluid negative for alcohols
   c. Blood negative for acidic, basic and neutral drugs

CAUSE OF DEATH

Blast and penetrating fragment injuries due to explosion.

GROSS DESCRIPTION

SKIN:

Multiple lacerations and perforations with irregular edges, searing and soot deposition

PLEURA: 200 mL blood right, 300 mL blood left, 2 deformed nails recovered from right pleural cavity, 5 x 2 inch threaded metal fragment recovered from left pleural cavity. Multiple smaller metal fragments recovered from both pleural cavities.

PERITONEUM: 100 mL blood, multiple metal fragments recovered

PERICARDIUM: Multiple lacerations.

HEART: 380 grams. Normal size and shape, coronary arteries within normal limits. Multiple lacerations of right and left ventricles. Laceration of aortic root.

AORTA: Laceration of aortic root. Multiple lacerations of lower abdominal aorta. Left iliac and femoral vessels fragmented.

NECK ORGANS: There are superficial penetrating fragment injuries on the anterior aspects of the neck with hemorrhage in the soft tissues adjacent to the wounds. Laryngeal cartilages, hyoid bone and cervical spine intact. Thyroid within normal limits. Airway free of blood and foreign material.

LUNGS: 930 grams combined. Multiple lacerations of upper lobes and right middle lobe. Parenchyma aerated with a few apical bullae.

LYMPH NODES: Within normal limits.

LIVER: 1650 grams. Multiple lacerations with partial pulpifaction of parenchyma.

GALLBLADDER: Contains bile. No stones.

SPLEEN: 130 grams. Multiple lacerations and partial pulpifaction.

PANCREAS: Usual external configuration and pale tan on cut section with the usual lobular architecture.

ADRENAL GLANDS: Within normal limits.

GI TRACT: There are fractures of the maxilla and mandible. The tongue is without injury. The pharynx and esophagus are unremarkable. The stomach is empty and there are multiple lacerations of the gastric wall. There are multiple lacerations and perforations of the mesentery, small intestine, and colon. The appendix is present.

KIDNEYS: 320 grams combined. There is hemorrhage in the perirenal soft tissues. The capsules strip with ease. The cortical surface is smooth. On cross section the cortices are of normal thickness and the pyramids and collecting systems are unremarkable. There are no stones or focal lesions. The ureters are intact.

BLADDER: Multiple lacerations of the wall.

GENITALIA: Lacerations of left scrotum with avulsion of left testicle and penis. Prostate unremarkable.

BRAIN & MENINGES: Brain absent. 950 grams of brain fragments submitted separately.

SKULL: Comminuted fractures of calvarium, skull base, and facial bones including mandible and maxilla.

PELVIS: Comminuted fractures of left ileum, left ischium, left pubic ramus. Separations of pubic symphysis and sacro-iliac joints.

RIBS: Perforations and fractures of anterior ribs and sternum.

VERTEBRAE: Within normal limits

EXTREMITIES: Amputations of right and left hands and forearms, Comminuted fractures of left proximal femur.

MICROSCOPIC SECTIONS: Heart, lung, liver

OTHER LAB PROCEDURES: Toxicology, Photography, X-ray, Microscopic Examination.

DISPOSITION OF EVIDENCE:

TOXICOLOGY: blood, vitreous, liver, bile, urine.

INVESTIGATOR: Clothing, recovered fragments.

MICROSCOPIC DESCRIPTION

HEART: Fragmentation and parenchymal hemorrhage

LUNGS: Lacerations and parenchymal hemorrhage. Focal emphysematous areas.

LIVER: Fragmentation and parenchymal hemorrhage.

SUMMARY:

This 34 year old white man died instantly from multiple blast and fragment injuries when a package exploded in his home.

CAUSE OF DEATH:

Blast and fragment injuries.

MANNER OF DEATH:

Homicide
